# Concretions in Large Intestine of a Swan

**Published:** 1881-07

**Authors:** 


### VI.—CONCRETIONS IN LARGE INTESTINE OF A SWAN.

At the post-mortem examination of a swan sent to the anatomical department from the Park, all the organs were apparently healthy except the large intestine. Here there was found a thick layer of hard crusts, with ragged edges. These solid masses were firmly united to the adjacent mucous membrane, and on forcible removal, portions of the layer were torn off from the intestine. The previous history of the swan was not communicated, nor did it appear that this abnormal intestinal condition bore any direct relation to the cause of death of the animal.

